# Expanding the CRISPR Toolbox in Zebrafish for Studying Development and Disease

**DOI:** 10.3389/fcell.2019.00013

**Published:** 2019-03-04

**Authors:** Kaili Liu, Cassidy Petree, Teresa Requena, Pratishtha Varshney, Gaurav K. Varshney

**Affiliations:** Functional and Chemical Genomics Research Program, Oklahoma Medical Research Foundation, Oklahoma City, OK, United States

**Keywords:** Cas12a (Cpf1), lineage tracing, base editors, zebrafish, disease models, CRISPR/Cas9

## Abstract

The study of model organisms has revolutionized our understanding of the mechanisms underlying normal development, adult homeostasis, and human disease. Much of what we know about gene function in model organisms (and its application to humans) has come from gene knockouts: the ability to show analogous phenotypes upon gene inactivation in animal models. The zebrafish (*Danio rerio*) has become a popular model organism for many reasons, including the fact that it is amenable to various forms of genetic manipulation. The RNA-guided CRISPR/Cas9-mediated targeted mutagenesis approaches have provided powerful tools to manipulate the genome toward developing new disease models and understanding the pathophysiology of human diseases. CRISPR-based approaches are being used for the generation of both knockout and knock-in alleles, and also for applications including transcriptional modulation, epigenome editing, live imaging of the genome, and lineage tracing. Currently, substantial effort is being made to improve the specificity of Cas9, and to expand the target coverage of the Cas9 enzymes. Novel types of naturally occurring CRISPR systems [Cas12a (Cpf1); engineered variants of Cas9, such as xCas9 and SpCas9-NG], are being studied and applied to genome editing. Since the majority of pathogenic mutations are single point mutations, development of base editors to convert C:G to T:A or A:T to G:C has further strengthened the CRISPR toolbox. In this review, we provide an overview of the increasing number of novel CRISPR-based tools and approaches, including lineage tracing and base editing.

## Introduction

Information gained from the study of model organisms is essential to our understanding of human development and disease. Replication of a mutant phenotype in a gene knockout (inactivation of a gene in an animal model) is considered to be the gold standard approach to support candidate gene predictions in humans. Zebrafish (*Danio rerio*) is uniquely suited to this approach and has become one of the fastest growing model organisms, useful for both basic and translational research (Bradford et al., 2017).

Zebrafish are an attractive alternative to mouse models because they give rise to a large number of progeny and are amenable to high-throughput mutagenesis and drug screening approaches (Kettleborough et al., 2013; Varshney et al., 2013; Varshney and Burgess, 2014; Gallardo et al., 2015). In addition, zebrafish fertilization is external, and their transparent larvae can be monitored throughout embryogenesis, providing unique accessibility to embryonic lethal mutations. The process of gene targeting in zebrafish is not as laborious as it is in mice, and the maintenance costs are 10X cheaper per animal (Varshney and Burgess, 2014). Importantly, zebrafish overcome an emerging technical issue in modeling disease pathology: many of the diseases studied today are multigenic, so disruption of a single gene may not produce a disease phenotype in any model system. However, combining genetic variants is straightforward in zebrafish, making it an ideal organism in which to model the functional consequences of multiple mutations. In addition, complementation studies in fish are relatively simple and allow for the direct testing of specific variants (not just knockouts) in a vertebrate system. The utility of zebrafish was further increased upon completion of the zebrafish genome-sequencing project (Howe et al., 2013); zebrafish and mammalian genes are highly conserved, and 70% of human disease genes have an ortholog in zebrafish (Howe et al., 2013). In zebrafish, many large-scale forward and reverse genetic screens have been performed using random mutagenesis methods - ENU or insertional mutagenesis (retroviral, Tol2, DS) - and the number of different genetic and molecular tools rapidly increased once the genome was sequenced (Amsterdam et al., 2011; Marquart et al., 2015; Quach et al., 2015; Seiler et al., 2015; Vrljicak et al., 2016). For decades, targeted gene knockouts were not possible in zebrafish, and its utility for validation studies of candidate genes was limited. This challenge was recently eliminated with the development of novel gene targeting approaches including ZFNs, TALENs, and CRISPR/Cas9 (Bedell et al., 2012; Jinek et al., 2012; Mali et al., 2013; Hsu et al., 2014; Varshney et al., 2015b); other techniques such as Structure Guided Nucleases (SGNs) have been shown to work for gene targeting in zebrafish but have not been adopted widely (Varshney and Burgess, 2016; Xu et al., 2016). With the transformative CRISPR/Cas9 approach, it is now possible to target any number of genes in an efficient and high-throughput manner (Gagnon et al., 2014; Shah et al., 2015; Varshney et al., 2015a). It is also possible to target multiple genes simultaneously; given that part of the zebrafish genome is duplicated, it is very useful in targeting 2 paralogs simultaneously (Jao et al., 2013).

CRISPR/Cas9 and other enzymes are not only being used to generate knockouts, introduce specific changes in the genome and repair mutant alleles, but are also being repurposed in other applications including transcriptional regulation, *in vivo* chromatin imaging, epigenome modulation, genome-wide knockout screens, etc. There are many reviews discussing the use of CRISPR-based approaches and the various technological developments in zebrafish (Varshney et al., 2015b; Li et al., 2016; Demarest and Brooks-Kayal, 2018). In this review we will focus on the latest development in expanding targeting coverage of gene targeting, base editing, transcriptional regulation, epigenome modulation, and lineage tracing.

## CRISPR-Mediated Targeted Mutagenesis in Zebrafish

In 2012, a joint team from Jennifer Doudna and Emmanuel Charpentier’s lab, and an independent team from Virginijus Siksnys’s lab demonstrated that Cas9 from *Streptococcus pyogenes* or *Streptococcus thermophilus* together with CRISPR RNA (crRNA) can be guided to a target site to cleave DNA *in vitro*. Early the following year, George Church and Feng Zhang’s labs utilized Cas9 from *Streptococcus pyogenes* and/or *Streptococcus thermophilus* to edit the genome in mammalian cells: they showed that single guide RNA (sgRNAs) can direct Cas9 to the target site to induce a double stranded break, which can then be repaired by either the non-homologous end joining (NHEJ) or homology-directed repair (HDR) pathways. An alternative repair pathway, microhomology-mediated end joining (aka Alt-EJ) (MMEJ; an error-prone repair mechanism that uses microhomologous sequences 5–25 bp in length) has also been shown to be activated by the double-stranded break induced by Cas9 (McVey and Lee, 2008; Ata et al., 2018). In the last 5–6 years, CRISPR-based genome editing tools have been used for many applications in a variety of cells, organisms and plants (Hsu et al., 2014). The use of simple and programmable CRISPR/Cas9 technology has completely transformed reverse genetics in zebrafish.

Zebrafish was the first vertebrate model used to demonstrate that CRISPR/Cas9 can efficiently edit the genome *in vivo* (Hwang et al., 2013) with up to 50% targeting efficiency. Another report demonstrated that CRISPR/Cas9 can be used to generate biallelic mutations in *gata5* and *etsrp*, and the observed phenotypes in injected embryos can phenocopy genetic mutants (Chang et al., 2013). Using a codon optimized version of Cas9 with nuclear localization signals, Jao et al. (2013) showed that Cas9 can efficiently induce biallelic mutations when Cas9 mRNA and sgRNA are injected into one-cell stage embryos. The authors further showed that up to five genes can be targeted simultaneously, and all showed phenotypes associated with each gene (Jao et al., 2013). It is evident from these initial reports that CRISPR/Cas9 is so efficient at inducing biallelic mutations that it allows for the generation of phenotypes in injected embryos similar to antisense morpholinos. Several strategies have been used to screen for phenotypes in the F_0_ generation in injected embryos; one such strategy used multiplexing to target multiple genes simultaneously and screen for phenotypes in F_0_. This approach was used to screen 48 genes and identify two novel genes involved in electrical-synapse formation (Shah et al., 2015). A similar strategy used a pool of four sgRNAs together with Cas9 protein to identify transcriptional regulators in cardiomyocytes; 50 candidate genes were screened and the role of *zbtb16a* in cardiac development was identified (Wu et al., 2018). Burger et al. (2016) demonstrated that the use of *in vitro* assembled Cas9 mCherry or EGFP fusion protein, and sgRNA together as a ribonucleoprotein complex can provide a visual readout for efficient microinjections for the analysis of mutant phenotypes in F_0_ generation. These mutants were termed CRISPR-mediated mutants or crispants (akin to morphants; Burger et al., 2016). While these approaches to screen candidate genes by analyzing the expected phenotypes in injected embryos are efficient, in most cases a stable mutant is required for phenotypic analysis of gene function. Data from Shawn Burgess’s lab targeting 89 genes show that genetic mutants can be generated with ∼28% germline transmission rates at a 99% success rate. This high germline transmission rate is four–fivefold higher than that of other targeting approaches such as ZFNs, and TALENs (Varshney et al., 2015a).

**FIGURE 1:**
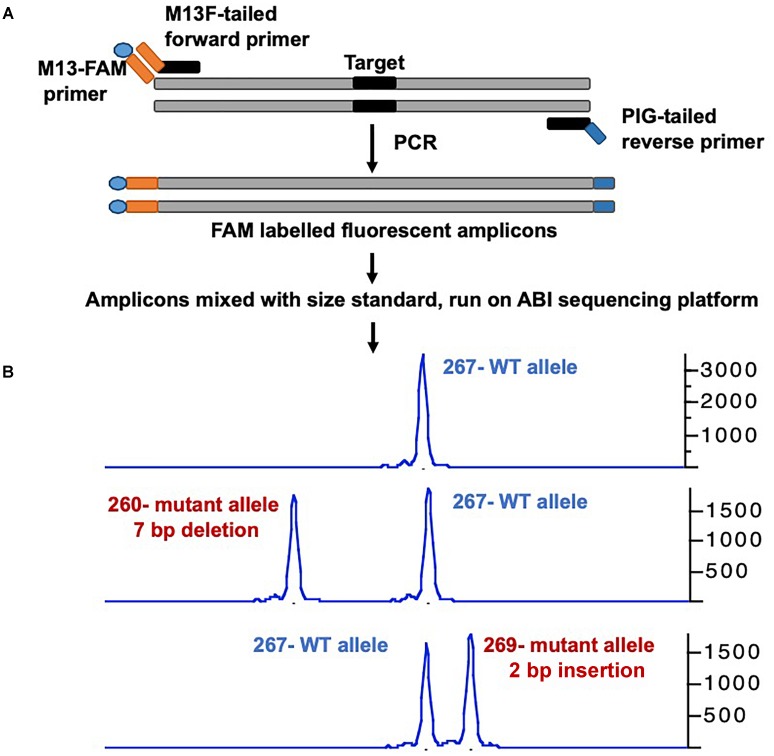
Overview of mutant identification method using fragment analysis approach. **(A)** Gene specific primers are designed covering the target site (amplicon size ranging 200–300 bp). Gene-specific forward primer contains M13F sequence at the 5′, and reverse primer has PIG-tail sequence at 5′ end. PCR is performed using gene specific primer set, and a third primer with M13F sequence labeled with FAM, resulting amplicons are fluorescently labeled. **(B)** Fluorescently labeled primers are mixed with size standard (e.g., ROX-400), and run on ABI capillary sequencer, and data is analyzed using gene mapper software. The output will have the size of amplicon, wild type allele will have only one size, while mutant allele will have two different sizes. The indel size can be determined by comparing the size of two alleles (WT vs. mutant).

Many groups have developed a streamlined workflow for generating mutants using CRISPR/Cas9 in a high-throughput manner (Gagnon et al., 2014; Varshney et al., 2015a, 2016a). The Burgess Lab addressed a few challenges in developing this workflow: First they developed a strategy to synthesize sgRNA by annealing two oligonucleotides that served as a template for *in vitro* transcription; this allowed for the synthesis of sgRNA in few hours with relatively low cost and is similar to the strategy was used by Gagnon et al. (2014). Secondly, the zebrafish genome is highly polymorphic, and it was predicted that this might cause multiple mismatches in the target sequence and prevent the sgRNA from binding efficiently. To address this, they sequenced the genome of the NHGRI-1 lab strain and identified more than 14 million variants. This data is available through UCSC genome browser track; while designing sgRNAs or PCR primers, variant regions of the genome can be avoided to maximize the success rate (LaFave et al., 2014). The third challenge they encountered was the identification of mutant alleles in a high-throughput manner. Several methods are currently used for the identification of mutants in zebrafish including DNA mismatch nuclease assays (Chang et al., 2013; Jao et al., 2013), restriction fragment length polymorphism (Hruscha et al., 2013) and sequencing (Gagnon et al., 2014; Varshney et al., 2015a; Burger et al., 2016), but none are amenable to high-throughput application. A method to determine the size of amplicons by fluorescent PCR was optimized to identify indels (Sood et al., 2013). This method uses three primers (gene-specific forward and reverse primers and a FAM-labeled primer) to amplify the regions around the target sites and resulting fluorescently labeled amplicons are mixed with a size standard (e.g., Rox400) to determine the amplicon size on ABI sequencing platform. This method can be applied in a high-throughput manner, and has resolution up to 1 bp (Figure 1) (Carrington et al., 2015; Varshney et al., 2015a).

Adopting CRISPR/Cas9 technology in a high-throughput manner for targeted mutagenesis has enabled geneticists to screen for large-numbers of genes with relatively modest resources, and generate disease models for ever-increasing candidate disease genes. The approaches have been widely applied: Pei et al. (2018) screened more than 200 candidate genes to identify genes involved in hair cell regeneration, and screens related to retinal regeneration or degeneration (Unal Eroglu et al., 2018) and several disease models including Niemman-Pick disease type C1;(Tseng et al., 2018), hearing disorders (Varshney et al., 2015a), congenital sideroblastic anemia with immunodeficiency, fevers and developmental delay (SIFD; (Giannelou et al., 2018), Mucolipidosis type IV (Li et al., 2017) have been performed. In zebrafish, phenotypes are generally studied in the F_2_ generation where homozygous embryos are generated by breeding two heterozygous (F_1_) lines over ∼6–7 months. It has been shown that phenotypes can be screened in the F_1_ generation in a non-Mendelian manner by inbreeding two founders, thus eliminating a generation and saving time (Varshney et al., 2015a). This could be an important strategy that may speed up the phenotypic screening of a large number of candidate disease genes. To generate knockouts, Cas9 is transiently expressed ubiquitously in one-cell stage embryos thus generating global knockouts, two independent studies have shown that Cas9 can also be expressed in a tissue-specific manner thus it is possible to inactivate genes in a specific tissues (Ablain et al., 2015; Yin et al., 2015). Yin et al. (2015) demonstrated that by using a heat-shock inducible and tissue specific promoters, the expression of Cas9 can be controlled both temporally and spatially [reviewed in (Li et al., 2016)]. They further characterized five U6 promoters to drive the expression of multiple guides thus adopting this approach for multiplex genome editing (Yin et al., 2015).

While CRISPR/Cas9 is an effective and simple tool for genetic manipulations, there are several concerns over its specificity as it has been shown to bind and edit unintended targets (e.g., Off targets) including inducing large deletions (Adikusuma et al., 2018). In zebrafish genetics, off-targets can easily be outcrossed away and a genotype-phenotype linkage must be established thus losing on-target activity by using Cas9 variants to achieve specificity should be considered. There are fewer studies in zebrafish that have tested the off-target effect in zebrafish. One such study detected off-target mutagenesis in only 1/25 off target sites in germline, another study showed off-target mutation rates from 1.1 to 2.5% (Hruscha et al., 2013; Varshney et al., 2015a). Many Cas9 variants such as Cas9-HF1, eSpCas9, evoCas9, HypaCas9, and others have been developed to increase the specificity of the Cas9 enzyme and thus reduce the off targets, however, these variant might also affect the on-target activities (Jamal et al., 2018).

We have summarized important CRISPR-based genome editing tools in Table 1. While SpCas9 can target multiple sites in the coding regions of the genome that is GC-rich, efforts are being made to expand target coverage by employing either orthologous Cas9 or evolving SpCas9 to identify different Protoacceptor Adjacent Motif (PAM) sequences.

**Table 1:** Commonly used tools for CRISPR-mediated genome editing.

Name	Description	URL	Reference
CRISPRScan	Tool to design Cas9/Cas12a targets.	http://www.crisprscan.org	Moreno-Mateos et al., 2015
CHOPCHOP	Tool to design Cas9, Cas9 variants, Cas12a targets, and genotyping primers. A custom PAM can also be selected.	http://chopchop.cbu.uib.no	Labun et al., 2016
ccTop	Target prediction tool for multiple Cas9 and Cas12a.	https://crispr.cos.uni-heidelberg.de	Stemmer et al., 2017
Cas-Designer	The most comprehensive tool to design Ca9, Cas9 variants, and Cas12a targets.	http://www.rgenome.net/cas-designer	Park et al., 2015
MENTHU	MENTHU (Microhomology-mediated End joining kNockout Target Heuristic Utility) is a tool for designing targets with microhomologies, to induce microhomology-mediated end-joining (MMEJ) deletions.	http://genesculpt.org/menthu	Ata et al., 2018
CRISPR-ERA	Cas9 target design tool for genome editing, repression, and activation	http://crispr-era.stanford.edu	Liu et al., 2015
CRISPResso 2	Webtool to analyze indels and base editing from the high-throughput sequencing data	http://crispresso.pinellolab.partners.org/	Clement et al., 2018
Cas-Analyzer	Online tool for analyzing indels from high-throughput sequencing data	http://www.rgenome.net/cas-analyzer/#!	Park et al., 2017
CRISPR-GA	CRISPR Genome Analyzer is a tool to identify indels from the next-generation sequencing data	http://crispr-ga.net/	Guell et al., 2014
CRISPRz	Database of validated sgRNA sequences in zebrafish	https://research.nhgri.nih.gov/CRISPRz/	Varshney et al., 2016b
inDelphi	Tool to predicts the indels resulting from microhomology-mediated end-joining (MMEJ) and non-homologous end-joining (NHEJ) repair.	https://www.crisprindelphi.design	Shen et al., 2018
FORECasT	Tool to predicts the indels generated by Cas9	https://partslab.sanger.ac.uk/FORECasT	Allen et al., 2018


## Engineered and Novel Nucleases to Expand the Targeting Coverage

Cas9 from *Streptococcus pyogenes* (spCas9) is the most popular and effective genome editing tool, and the sequences recognized by *SpCas9* are limited by the specific and simple PAM (5′-NGG-3′) requirement (Jinek et al., 2012). However, this specific PAM sequence may not be available near the target of interest. To expand the targeting coverage, researchers have identified additional, naturally occurring CRISPR nucleases that may have different PAM requirements. Additionally, spCas9 was engineered to recognize other PAM sequences, expanding the targeting coverage and allowing them to be used in orthogonal applications. These newly identified CRISPR nucleases may also address the challenge of delivering the large size of spCas9 (1,368 aa) as they may be smaller; they may also provide a homology template for *in vivo* therapeutic applications (Mout et al., 2017).

### Orthologous Cas9

Many of the smaller-sized Cas9 nucleases discovered in different species can recognize different PAM sequences and varied lengths of target sequences for *in vivo* genome editing (Table 2): the *Staphylococcus aureus* Cas9 (SaCas9, 1053 aa) is not only small in size, but also uses a different complex PAM (NNGRRT; (Muller et al., 2016). Many other Cas9 nucleases from different bacterial species are being used for *in vivo* genome editing: Cas9 from *Neisseria meningitidis* (NmCas9) requires NNAGAAW PAM; Cas9 from *Streptococcus thermophilus* (St1Cas9, 1121 aa and St3Cas9, 1388 aa) require NNAGAAW and NGGNG PAMs, respectively; Cas9 nuclease from *Campylobacter jejuni* (CjCas9, 984 aa) recognizes a 22-nt target sequence with NNNVRYAC and NNNNRYAC PAM (Kim E. et al., 2017).

**Table 2:** Summary of Cas orthologs and variants.

CRISPR Cas orthologs or variants	Recognized PAM	Target length	Use in zebrafish	Reference
*Streptococcus pyogenes* Cas9 (SpCas9)	NGG	19 or 20 nt	Yes	Jinek et al., 2012; Cong et al., 2013
*Streptococcus pyogenes* Cas9 Variant VQR *(SpCas9 VQR)*	NGAN, NGNG	19 or 20 nt	Yes	Kleinstiver et al., 2015b
*Streptococcus pyogenes* Cas9 Variant EQR *(SpCas9 EQR)*	NGAG	19 or 20 nt	Yes	Kleinstiver et al., 2015b
*Streptococcus pyogenes* Cas9 Variant VRER *(SpCas9 VRER)*	NGCG	20 nt	Yes	Kleinstiver et al., 2015b
*Streptococcus pyogenes* Cas9 Variant D1135E *(SpCas9 DE)*	NAG	20 nt	Not tested	Kleinstiver et al., 2015b
*Streptococcus pyogenes* Cas9 Variant QQR1 (SpCas9 QQR1)	NAAG	20 nt	Not tested	Anders et al., 2016
*Streptococcus pyogenes variant TLIKDIV (x*Cas9 3.7)	NG, NNG, CAA, GAT, GAA	20 nt	Not tested	Hu et al., 2018
*Streptococcus pyogenes NG variant (SpCas9NG)*	NGA, NGT, NG	20 nt	Not tested	Nishimasu et al., 2018
*Staphylococcus aureus* Cas9 (SaCas9)	NNGRRT	20–24 nt	Not tested	Ran et al., 2015
*Staphylococcus aureus* KKH Cas9 variant (SaCas9 KKH)	NNNRRT	21 nt	Yes	Kleinstiver et al., 2015a
*Streptococcus thermophilus 1* Cas9 (St1Cas9)	NNAGAAW (W = A or T)	20 nt	Not tested	Muller et al., 2016
*Streptococcus thermophilus*3 Cas9 (St3Cas9)	NGGNG	20 nt	Not tested	Glemzaite et al., 2015
*Neisseria meningitidis* Cas9 (Nm or NmeCas9)	NNNNGMTT (M = A or C)	23–24 nt	Not tested	Hou et al., 2013; Fonfara et al., 2014
*Campylobacter jejuni* Cas9 *(CjCas9)*	NNNVRYAC NNNNRYAC NNNVRYM (R = A or G) (Y = C or T) (M = A or C)	22 nt	Not tested	Kim E. et al., 2017; Yamada et al., 2017
*Francisella novicida* Cas9	NGG	22 nt	Not tested	Fonfara et al., 2014; Hirano et al., 2016
*Francisella novicida* Cas9 RHA variant	YG (Y = C or T)	22 nt	Not tested	Hirano et al., 2016
*Treponema denticola* Cas9 *(TdCas9)*	NAAAAN	20 nt	Not tested	Esvelt et al., 2013
*Streptococcus macacae Cas9 (SmacCas9)*	NAAN	20 nt	Not tested	Jakimo et al., 2018
*Streptococcus canis (ScCas9)*	NNG	20 nt	Not tested	Chatterjee et al., 2018
*Streptococcus canis (ScCas9) ΔLoopΔKQ variant*	NNGA, NGG	20 nt	Not tested	Chatterjee et al., 2018
*Acidaminococcus* Cas12a (AsCas12a/Cpf1)	TTTV (V = A or C)	23 or 24 nt	Yes	Zetsche et al., 2015
*Lachnospiraceae* Cas12a (LbCas12a/Cpf1)	TTTV (V = A or C)	23 or 24 nt	Yes	Zetsche et al., 2015
*Francisella Cas12a (FnCas12a/Cpf1)*	TTN, KYTV (K = G or T) (Y = C or T) (V = A or C)	23 or 24 nt	Yes	Zetsche et al., 2015; Tu et al., 2017
*Moraxella Cas12a (MbCas12a/Cpf1)*	TTN	23 or 24 nt	Not tested	Zetsche et al., 2017
*AsCas12a, LbCas12a, FnCas12a, and MbCas12a* RR variants	TYCV, TWTV (W = A or T) (V = A or C) (Y = C or T)	23 or 24 nt	Not tested	Gao et al., 2017; Nishimasu et al., 2017; Toth et al., 2018
*AsCas12a, LbCas12a, FnCas12a, and MbCas12a* RVR variant	TATV (V = A or C)	23 or 24 nt	Not tested	Gao et al., 2017; Nishimasu et al., 2017; Toth et al., 2018


### Engineered Cas9 Variants

Most of the orthologous Cas9 nucleases have long and complex PAM requirements that will limit the targeting range because they will occur less frequently in genomes. An alternative strategy to expand PAM specificity would be to engineer the SpCas9 to recognize other PAMs. Kleinstiver et al. (2015b) engineered SpCas9 based on the crystal structure of the enzyme, and the mutated Cas9 was tested for its ability to recognize different PAM sites. Engineered SpCas9 variants VRER (D1135V/G1218R/R1335E/T1337R) recognizing NGCG PAM, VQR (D1135V/R1335Q/T1337R) recognizing NGAN or NGNG PAM, and EQR (D1135E/R1335Q/T1337R) variants recognizing NGAG PAM were generated. All of these SpCas9 variants were able to target sequences that were not targetable by wild-type SpCas9 in human cells, but only the VQR variant was able to target sites with NGAG PAMs (20–43% efficiency in zebrafish; (Kleinstiver et al., 2015b). The efficiency of the VQR was further validated by showing its ability to target *tyr* and *EGFP* loci with 50 and 70% efficiency, respectively. Zebrafish codon-optimized versions of VQR and EQR SpCas9 generated by Shawn Burgess’ lab are also available from Addgene (Varshney et al., 2016a).

As described above, the majority of the Cas9 orthologs or variants have complex PAM requirements, and the frequency of these targets in the genome is limited. To circumvent this challenge, David Liu’s lab used phage-assisted continuous evolution (PACE) to isolate 14 evolved SpCas9 variants (xCas9 3.0–3.13); one such variant (xCas9 3.7) was able to recognize a broad range of PAM, including NG, NNG, CAA, GAT, and GAA (Hu et al., 2018). The xCas9 3.7 variant was able to cleave multiple PAMs at much higher frequency than wild-type SpCas9: GAA and GAT PAM showed ∼5-fold, NGT ∼4.5-fold and NGC 2.1-fold efficiencies. Another variant, xCas9 3.6, showed the second-best editing efficiencies at fewer PAMs (Hu et al., 2018). SpCas9 was further engineered to generate a variant called SpCas9-NG that has a relaxed preference for the third nucleobases in the NGG PAM (Nishimasu et al., 2018). This variant had seven residues mutated (R1335V/L1111R/D1135V/G1218R/E1219F/A1322R/T1337R) in SpCas9; was capable of cleaving NGA, NGT, and NGG PAMs with more than 20% editing efficiency; and showed lower activity at NGC PAM. A comparison of editing efficiencies showed that spCas9-NG had higher editing efficiencies at NGA, NGT, and NGG sites, and xCas9 failed to edit NGC PAM targets (Nishimasu et al., 2018).

Similarly, *Staphylococcus aureus* Cas9 (SaCas9) was also modified using a molecular evolution strategy to recognize NNNRRT PAMs. This variant of SaCas9 is known as KKH SaCas9 (variant E782K/N968K, R1015H) and can further increase the SaCas9 targeting range by two–fourfold (Kleinstiver et al., 2015a). The KKH SaCas9 variant was able to recognize five independent targets in different genes with 10–90% efficiency, thus expanding targeting coverage further in zebrafish (Feng et al., 2016).

The Cas9 nuclease from *Francisella novocida* (FnCas9) is one of the largest nucleases identified thus far (1629 aa) and recognizes NGG PAM similar to SpCas9, but has failed to generate indels in mammalian cells. It is possible that microinjecting mouse zygotes with FnCas9 protein and a guide RNA ribonucleoprotein complex may induce target-specific indels; a variant of FnCas9 (E1369R/E1449H/R1556A) called RHA FnCas9 could recognize YG PAM (Hirano et al., 2016).

Recently, a homolog of *SpCas9* in *Streptococcus macacae* (*SmacCas9*) has been described to recognize the 5′-NAAN-3′ PAM. A variant of *SmacCas9* (iSpy-macCas9) was engineered to maintain its specificity for adenine dinucleotide PAM while showing higher genome editing efficiency *in vivo* (Jakimo et al., 2018). An orthologous Cas9 protein from *Streptococcus canis* (ScCas9) with 89.2% sequence similarity to wild-type SpCas9 has also been characterized and shown to prefer a more minimal NNG (Chatterjee et al., 2018). An engineered version of ScCas9 (ΔLoop ΔKQ) not only cleaves NGG PAM but also recognizes and edits NNGA PAM at a comparable rate, but it edits other NNGN PAMs with reduced efficiency. All of these engineered and orthologous Cas9 proteins have significantly expanded the targeted coverage.

### CRISPR/Cas12a (Cpf1)

**FIGURE 2:**
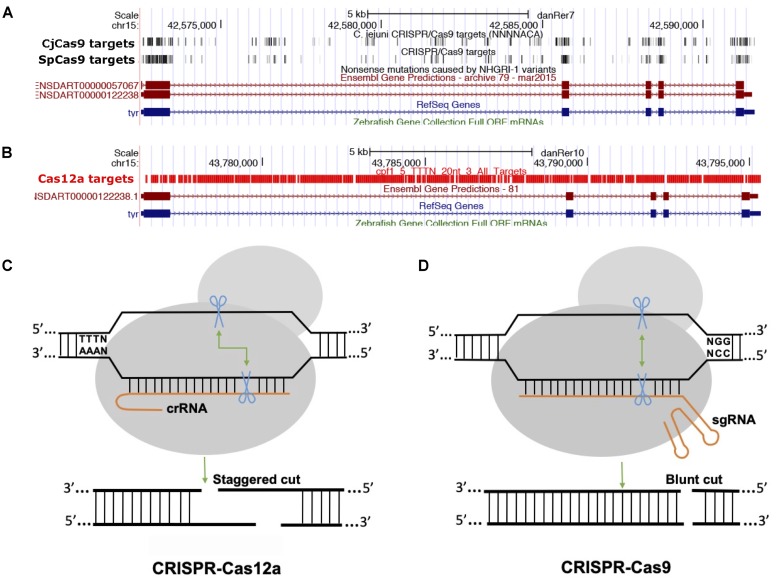
Comparison between Cas9 and Cas12a. **(A)** Screenshot of UCSC genome browser tracks showing predicted target sequences in *tyr* gene for CjCas9, and SpCas9. The targets for both Cas9 nucleases are enriched in coding exons, **(B)** while Cas12 target sequences are enriched mostly in intronic sequences. **(C,D)** Comparison of Cas12a and Cas9, Cas12 a generates a staggered cut, Cas9 induces a blunt end cut.

The majority of class 2 and type II nucleases and their engineered versions described earlier have preference for GC-rich PAMs that limits the targeting of AT-rich sequences, for example most of the non-coding genome in zebrafish is AT-rich (Howe et al., 2013). Another class 2 and type V family of nucleases, originally described as Cpf1 and later renamed Cas12a (Shmakov et al., 2017), was discovered as an alternative effective genome-editing tool (Zetsche et al., 2015). Cas12a is different from SpCas9 in many ways (Figure 2): (i) Cas12a recognizes T-rich PAM located at the 5′ end of the target DNA sequence, (ii) Cas12a is guided by a single CRISPR RNA (crRNA) that is shorter than that of SpCas9 and does not require *trans*-acting crRNA (tracrRNA), (iii) Cas12a uses an ∼23 nt target sequence, (iv) Cas12a induces a double-stranded break in the target sequence via a staggered cut and ∼18 nt distal to PAM, generating a 4–5 nt 5′ overhang, and (v) Cas12a has both DNAse and RNase activity; therefore it is capable of processing its own CRISPR array (Fonfara et al., 2016). As of now, 32 Cas12a orthologs have been described, and their genome-editing potential was screened. Cas12a from *Francisella novicida* (FnCas12a), *Acidaminococcus* sp. BV3L6 (AsCas12a), and *Lachnospiraceae* bacterial ND2006 (LbCas12a) exhibited robust editing in human cells, plants and many other model organisms, including zebrafish (Zetsche et al., 2015). Additionally, four other Cas12a orthologs [*Thiomicrospira* sp. Xs5 (TsCas12a), *Moraxella bovoculi* AAX08_00205 (Mb2Cas12a), *Moraxella bovoculi* AAX11_00205 (Mb3Cas12a), and *Butyrivibrio* sp. NC3005 (BsCas12a)] have been shown to induce indels in human cells, although only Mb3Cas12a was able to induce indels at a rate comparable to AsCas12a and LbCas12a (Zetsche et al., 2017). AsCas12a and LbCas12a use TTTV PAM, while FnCas12a and Mb3Cas12a recognize the less-restrictive TTN and NTTN PAMs, respectively. FnCas12a also has been show to target sequences with KYTV PAM preference in mammalian cells (Tu et al., 2017). The Cas12a nucleases were further engineered by introducing mutations S542R/K607R and S542R/K548V/N552R to generate AsCas12aRR and AsCas12RVR variants, and G532R/K592R and G5323R/K538V/Y542R to generate LbCas12a RR or RVR variants, which can recognize non-canonical PAMs such as TYCV, TWTV, and TATV PAMs. Use of Cas12a in editing the zebrafish genome is not as straightforward as editing using SpCas9. Cas12a mRNA and crRNA targeting the *tyr* locus do not induce any indels in zebrafish at optimal temperature (28°C) (Watkins-Chow et al., 2017). Further optimization revealed that Cas12a crRNAs are degraded rapidly after injection in one-cell stage embryos; however, LbCas12a-crRNA ribonucleoprotein (RNP) complex can protect crRNA from degradation and can efficiently induce indels at a rate comparable to that of SpCas9 in zebrafish (Moreno-Mateos et al., 2017). LbCas12a is more effective in inducing indels than AsCas12a, and AsCas12a activity is temperature dependent in zebrafish. Heat shocking embryos after injection for 4 h at 34°C significantly increased the mutagenic activities for AsCas12a and LbCas12a nucleases. (Moreno-Mateos et al., 2017) LbCas12a has been shown to achieve higher homology-directed repair compared to SpCas9. LbCas12a-mediated HDR is most efficient when an ssDNA donor template that is complementary to the target strand is provided (SpCas9 favors the non-target strand). Cas12a nucleases have expanded the targeted coverage many fold, which will help target non-coding regions that are AT rich in zebrafish (Moreno-Mateos et al., 2017).

#### Base Editing Tools

The majority of genetic diseases are caused by point mutations (single or multiple) that result in amino acid substitutions which generate non- or partially functional proteins. Studying these mutations in a model organism using gene knockout technology may not completely mimic the mutations found in human patients. Creating these mutations in zebrafish has been challenging: a targeted knock-in mutant is achieved via homologous recombination by delivering sgRNA and Cas9 together with either a single-stranded oligonucleotide or a donor plasmid containing the left and right homologous arms. Several strategies have been developed for introducing specific changes using knock-in technologies (Prykhozhij et al., 2018; Tessadori et al., 2018; Zhang et al., 2018), and reviewed in many publications (Albadri et al., 2017).

However, the success rate of homology-directed repair (HDR) is extremely low, so introducing specific changes in the genome has been difficult because repair machinery tends to favor non-homologous end joining repair. Moreover, HDR requires the delivery of donor template to the target cells and precise repair of the genomic sequence. Recently, Jeffery Essner’s lab described an optimized targeted knock-in strategy, called GeneWeld, in which they developed a series of donor plasmids for gene tagging [pGTag-plasmids for Gene Tagging (58)]. This strategy is based on the targeting of multiple genomic loci using donor plasmids with short homology arms (24–48 bp), and can be used to integrate cargos up to 2 kb in zebrafish with high efficiency [up to 50% germline transmission (58)]. This technology should allow for maximal integration of fluorescent tags.

**FIGURE 3:**
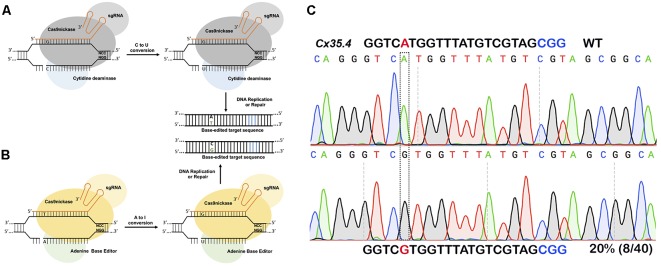
Single nucleotide substitution using base editors. **(A)** Cytidine deaminase fused to nickase Cas9 converts cytosine to thymine to guanine to adenine within a targeting window. **(B)** Adenine base editor converts adenine to inosine that is recognized as guanine during DNA replication or repair thus converting A to G or C to T. **(C)** *In vivo* substitution of adenine to guanine using ABE7.10 base editor in zebrafish. sgRNA targeting cx35.4 gene was injected in 1-cell stage zebrafish embryos, DNA from a pool of four injected embryos was sequenced, and 20% of the clones carrying the desired A to G substitution.

For introducing point mutations, recent progress in CRISPR-mediated base editing allows for the introduction of point mutations (conversion of G-C base pairs to A-T base pairs or vice-versa) without inducing a double-stranded break (Figure 3; Komor et al., 2016; Gaudelli et al., 2017).

The first-generation CRISPR base editor (BE1) uses catalytically inactive Cas9 (dCas9) fused with cytidine deaminase enzyme encoded by the rat APOBEC1 gene (Komor et al., 2016). The cytidine deaminase enzyme converts cytosine bases into uridine, which are then read as thymine during replication. The result is a conversion of cytosine to thymine that occurs within the five-nucleotide window. The second-generation base editor (BE2) is fused with a uracil glycosylase inhibitor (UGI) that prevents excision of uracil during repair; BE2 has marginally higher activity compared to BE1 but does induce indel formation because it contains dCas9not. To further improve editing efficiency, the catalytic His residue at position 840 (which nicks the non-edited strand to mimic newly synthesized DNA) was restored to create BE3, the most widely used base editor. BE3 is the most efficient of the three base editors, and may also induce indels due to its nicking capabilities (Komor et al., 2016).

A new version of BE3 – HF-BE3 – was developed by incorporating mutations in Cas9 known to increase specificity and decrease off-target editing; in practice HF-BE3 appears to have lower on-target editing efficiency (Rees et al., 2017). Delivering the BE3- ribonucleoprotein complex (RNP) results in more robust editing than using plasmid-mediated delivery. This efficiency of BE3 RNP was further tested in zebrafish to generate specific point mutations targeting the tyrosinase locus: two of the three BE3:sgRNA RNP complexes were able to induce substantial point mutations *in vivo*, with 4–5% editing efficiency (Rees et al., 2017).

David Liu’s group continued their effort toward refining and improving the base editors. They engineered the next generation base editors (BE4) to increase the base conversion efficiency by 50%. BE4 editors have extended (32 aa) rAPOBEC1-Cas9n and Cas9n-UGI linker (9 aa), and fusion of an additional UGI to the C terminus with another 9-amino acid linker. The BE4 base editor was further refined by adding the bacteriophage Mu protein Gam, which binds to double-stranded breaks and reduces indel formation to less than 1.5%; this modified base editor is called BE4-Gam (Komor et al., 2017). To increase APOBEC1 expression, ancestral sequence reconstruction using 468 homologs of APOBEC1 was performed, and two ancestors (Anc689 and Anc687) were selected (Koblan et al., 2018). Codon-optimized bipartite NLS were added at the N- and C-termini, similar to BE4max, to create the AncBE4max variant that showed improved editing at multiple loci (Koblan et al., 2018).

In zebrafish, cytidine deaminase fused with Cas9 nickase was able to induce sequence-specific single base mutations from ∼9 to 28% efficiency at multiple loci with a low number of indels (Zhang et al., 2017). Authors targeted *tyr* gene causal gene for human ocular albinism (OA) and oculocutaneous albinism (OCA). A mutation p.P301L in the *tyr* gene has been identified in OCA patients. While they were not able to convert proline to leucine, proline was converted to three other amino acids: serine, alanine or threonine. Edited embryos showed the loss of pigmentation in the eyes of injected embryos. Five other targets tested also converted cytosine to thymine with varying efficiency (Zhang et al., 2017). While the BE system works in zebrafish, the efficiency is low compared to knockouts, and further optimization is required to improve editing efficiency.

Similar to the BE system, the “Target-AID system” was developed by Japanese researchers. The Target-AID system is composed of nuclease-dead Cas9 or Cas9 nickase fused with activation-induced cytidine deaminase (AID) encoded by the PmcDA1 gene from sea lamprey. Target-AID can also induce cytosine to thymine conversion within a five-nucleotide window. The target-AID system was used in zebrafish to introduce premature stop codons (TAG or TAA) by converting cytosine to thymine. Two genes, chordin (*chd*) and one-eyed pinhead (*oep*) were targeted using this strategy, and the introduction of premature stop codons phenocopies the known genetic mutants (Tanaka et al., 2018).

Recently, another base editor (eA3A-BE3) fused with an engineered human APOBEC3A (eA3A) domain was shown to deaminate cytidines in a more controlled manner, and function according to a TCR > TCY > VCN (V = G, A, C, Y = C, T) hierarchy (Gehrke et al., 2018). The new base editor variant has shown comparable activities on cytidines in TC motifs, with reduced or no significant editing on cytidines in other motifs. Furthermore, eA3A-BE3 has shown low undesirable bystander mutations compared to other versions (Gehrke et al., 2018).

Existing cytosine deaminase base editors can target bases located between the 4th and 8th position in the target sequence. To expand the targeting window, a new base editor for programming larger C to U (T) scope (BE-PLUS) was developed. This new editor utilizes the SunTag system (Jiang et al., 2018); SunTag contains multiple copies of GCN4 peptide (each consisting of 19 residues) which is recognized by a single chain variable (scFV) antibody. BE-PLUS contains three components: nickase Cas9 fused at the C-terminus to 10 copies of GCN4 peptide (SunTag), scFv-APOBEC-UGI-GB1, and sgRNA. BE-PLUS induced fewer C-T conversions at positions 4–8, but converted C-T at 9–16 positions more efficiently. However, at positions 4–8, BE-PLUS was as efficient as the previously described BE3 (Jiang et al., 2018).

Third- and fourth-generation base editors (BE3 and BE4Gam) were further optimized by codon optimizing Cas9, as well as by adding a FLAG tag and NLS at the N-terminus. These modified base editors were shown to improve C-T conversion up to 50-fold compared with the original BE3 or BE4 base editors (Zafra et al., 2018). A novel method - CRISPR-SKIP - has been shown to program exon skipping by mutating splice acceptor sites using cytidine deaminase (Gapinske et al., 2018). The CRISPR-SKIP webtool can identify exons that can be skipped using this method, and it currently supports BE3, VQR-BE3, VRER-BE3, and SaKKH-BE3 variants (Gapinske et al., 2018). Michael Bassik’s lab developed a novel base editor CRISPR-X, which uses an RNA aptamer (MS2) fused to sgRNA to recruit the cytidine deaminase to the target site and induce somatic hypermutation within a 100 bp window. This is a powerful approach for protein engineering because it can generate a diverse population of alleles that could be useful for directed evolution (Hess et al., 2016).

### Adenine Base Editors

Cytidine deaminase-based base editors convert C-T or G-A; there are no natural enzymes that can convert A-G or T-C. To address this problem, David Lius’ lab developed an adenine base editor (ABE) to modify adenine bases. The existing adenosine deaminase TadA/ADAR enzymes do not act on double-stranded DNA. Using phage-assisted continuous evolution (PACE), multiple rounds of directed evolution led to the identification of *Escherichia coli* TadA that can use DNA as a substrate. The ABE consists of a nickase Cas9 fused with a heterodimer of wild-type TadA and engineered TadA enzymes, guided by sgRNA to the target site. Engineered TadA converts adenine (A) to inosine (I) on the DNA target; inosine is recognized as guanine during DNA repair or replication, thus converting adenine (A) to guanine (G) or thymine (T) to cytosine (C). Of the Liu lab’s multiple versions from ABE 0.1 to ABE 7, ABE7.10 has been shown to convert AT to GC with approximately 50% efficiency in mammalian cells. The ABE7.10 variant converts bases at position 4 to 7, and ABE7.8 or ABE7.9 variants convert bases at positions 4–9. The ABE7.10 variant was further optimized to generate a new variant, ABEmax, by replacing SV40NLS to codon-optimized bipartite NLS at both the N- and C-termini. Modified ABEmax increases the base substitution rate from ∼1.5- to 2-fold without changing the editing window; however, the rate of indels slightly increased.

Both cytidine deaminase and adenine deaminase enzymes are further fused with different variants such as VQR, VRER, SaCas9KKH, and newly evolved Cas9 such as xCas9, iSpy-macCas9, and SpCas9-NG (Kim Y. B. et al., 2017; Hu et al., 2018). Thus, both types of base editors will provide coverage to change all four bases in a targeted manner. As the new variants and new orthologs of nucleases evolve the targeting coverage will further expand covering all of the pathogenic variants. Tables 3, 4 summarizes the different base editing resources, targeting range of each base editors mentioned above, respectively.

**Table 3:** Resources for base editing.

Resource	Description	URL	Reference
BE-Analyzer	NGS data analysis tool to identify based editing induced events.	http://www.rgenome.net/be-analyzer/	Hwang et al., 2018
BE-Designer	Guide-RNA design tool for base editing.	http://www.rgenome.net/be-designer/	Hwang et al., 2018
BEEP	Command line program to assess CRISPR-mediated base editing efficiency from Sanger sequencing ab1 files.	https://github.com/mitmedialab/BEEP	Chatterjee et al., 2018
CRISPR-SKIP	A tool to design induce exon skipping by base editing.	http://song.igb.illinois.edu/crispr-skip/	Gapinske et al., 2018
CRISPResso 2	Tool to analyze base editing events from next generation sequencing data.	http://crispresso.pinellolab.partners.org/	Clement et al., 2018
EditR	Tool to estimate base editing by Sanger sequencing.	http://baseeditr.com/	Kluesner et al., 2018
iSTOP	Database of sgRNAs for generating STOP codons using base editing.	http://www.ciccialab-database.com/istop	Billon et al., 2017
Beditor	Tool to design genome-wide sgRNA for base editing.	https://github.com/rraadd88/beditor	Dandage et al., 2018


**Table 4:** Summary of editing windows by base editors.

Base editor (s)	Editing window	Use in zebrafish	Reference
**Cytosine deaminase**			
SpCas9-BE1, SpCas9-BE2, SpCas9-BE3, SpCas9-BE4 SpCas9-BE4max, and SpCas9-BE4-Gam	4–8	Yes	Komor et al., 2016, 2017; Kim Y. B. et al., 2017
SpCas9VQR-BE3	4–11	Yes	Kim Y. B. et al., 2017
SpCas9VRER-BE3	3–10	Not tested	Kim Y. B. et al., 2017
SpCas9YE1-BE3	4–7	Not tested	Kim Y. B. et al., 2017
SpCas9YE2-BE3, SpCas9YEE-BE3, SpCas9YEE-BE3	5–6	Not tested	Kim Y. B. et al., 2017
SaCas9-BE3, SaCas9-BE4, SaCas9KKH-BE3	3–12	Not tested	Kim Y. B. et al., 2017
xCas9-BE3	4–8	Not tested	Hu et al., 2018
SpCas9 Target-AID	2–4	Yes	Nishida et al., 2016
SpCas9-NG Target-AID	2–4	Not tested	Nishimasu et al., 2018
SpCas9-BE-Plus	4–16	Not tested	Jiang et al., 2018
SpCas9 eA3A-BE3, A3A-BE3	4–8	Not tested	Gehrke et al., 2018
CRISPR-X	-50 bp to +50 bp relative to PAM	Not tested	Hess et al., 2016
Cas12a (Cpf1)-BE	8–13	Not tested	Li et al., 2018
**Adenine base editors**			
ABE7.9	4–9	Not tested	Gaudelli et al., 2017
ABE7.10	4–8	Not tested	Gaudelli et al., 2017
xCas9 ABE	4–8	Not tested	Hu et al., 2018
SpCas9-VQR ABE	4–8	Not tested	Yang et al., 2018
SaCas9-KKH ABE	6–12	Not tested	Yang et al., 2018
ScCas9-ABE7.10	4–8	Not tested	Chatterjee et al., 2018


## Transcriptional Modulation and Epigenome Editing

CRISPR/Cas9 has also been repurposed to modulate transcription and manipulate the epigenome. In order to apply the CRISPR system beyond inducing a double stranded break, the DNA cleavage activity of Cas9 nuclease must be inactivated. Cas9 from *Streptococcus pyogenes* (SpCas9) contains two nuclease domains – a RuvC-like domain and a HNH domain – both of which are required to induce a double stranded break (Jinek et al., 2012). Introducing mutations in the catalytic residues of both nuclease domains (D10A, H840A) will create a catalytically inactive version of the Cas9 (dCas9; Qi et al., 2013). Using sgRNA, dCas9 can be recruited to a specific target without inducing a DNA break. To modulate transcription, dCas9 was first fused with transcriptional activators (VP64, a synthetic tetramer of the Herpes Simplex Viral Protein or p65 a transcription factor activation domain) or transcriptional repressors [KRAB, a Kruppel-associated box and the transcriptional repressor of Kox1, or 4X mSin Interaction Domain (SID; Konermann et al., 2015)]. These fusion proteins result in transcriptional activation (CRISPRa) or repression (CRISPRi) when targeted to the regulatory or coding regions of the gene.

Both CRISPRa and CRISPRi have been shown to work in modulating transcription of the target genes in zebrafish (Long et al., 2015). Two genes required for otic placode induction (*fgf8a* and *foxi1*) were targeted to demonstrate the utility of CRISPRa and CRISPRi in zebrafish. When sgRNAs targeting *fgf8a* coding regions were co-injected with dCas9-KRAB (CRISPRi) fusion protein, the expression of *fgf8a* was reduced at 11 hpf, and smaller otic vesicles were observed at 32 hpf (Long et al., 2015). Similarly, when dCas9-VP160 (CRISPRa) together with either sgRNAs targeting *fgf8a* or *foxi1* were injected in one-cell stage embryos, the expression of *fgf8a* and *foxi1* was increased and the resulting animals showed enlarged otic vesicles.

The dCas9 was also fused with putative Eve repressor domain of zebrafish *Evx1*, and the resulting dCas9-Eve fusion together with three sgRNAs targeting sequences upstream of all zinc finger transcription factors (*zfnl1s*) were used to inhibit the transcription of the *znfl1* in zebrafish (Dong et al., 2017). Decreased expression of *znfl1* disrupts the formation of the posterior neuroectoderm in zebrafish gastrula, and the phenotype perfectly phenocopies that generated by the anti-sense morpholino (Dong et al., 2017).

In zebrafish it has been shown that mutants generated by targeting mutagenesis techniques, genetic compensation or transcriptional adaptation could all trigger the upregulation of related genes and compensate for the loss of the targeted gene. Such upregulation and compensation were not observed when antisense morpholinos were used, suggesting that downregulation of target genes using CRISPRi could be an alternate tool to study gene function.

Additionally, catalytically inactive Cas9 has been fused to various epigenetic effectors such as the catalytic core of the human acetyltransferase p300 which catalyzes acetylation of histone H3 lysine 27 (Hilton et al., 2015), histone demethylase (Kearns et al., 2015), histone deacetylase (HDAC) (Kwon et al., 2017) and many others (reviewed in Lau and Suh, 2018).

## Lineage Tracing Using CRISPR/Cas9

A fundamental goal in developmental biology is to determine the origin of different cell types and tissues, and to establish their relationship in complex organisms. Lineage tracing is one method employed by developmental biologists to study the origin of cell types: techniques include dye based markers, nucleotide pulse-chase analysis, transplantation, sequencing somatic mutations, Cre-Lox and FLP-FRT based methods (reviewed in Kretzschmar and Watt, 2012). These methods can efficiently label cells at a single time-point to study large numbers of clonal populations in a complex animal, however, a detailed lineage tree over time cannot be reconstructed; understanding how cells change over the time will help determine the mechanisms of disease progression. Recently, the CRISPR/Cas9 mediated genome editing technique was used to generate genetic scars (indels) in the genome which serve as genetic barcodes for use in the reconstruction of cell lineages in developing animals or adults. Using this principle, many innovative approaches have been developed including genome editing of synthetic target arrays for lineage tracing (GESTALT; McKenna et al., 2016), lineage tracing by nuclease-activated editing of ubiquitous sequences (LINNAEUS; Spanjaard et al., 2018), ScarTrace (Alemany et al., 2018), and memory by engineered mutagenesis with optical *in situ* readout (MEMOIR; Frieda et al., 2017). CRISPR based lineage tracing is being adopted in multiple model organisms including zebrafish (Schmidt et al., 2017; Kalhor et al., 2018; Raj et al., 2018; Spanjaard et al., 2018). GESTALT, first applied to the understanding of the origin of organ development in zebrafish, was developed in the labs of Jay Shendure and Alex Schier. The Schier Lab engineered 10 different target sequences (unique barcodes in the 3′ UTR of DsRed) that are not found in the zebrafish genome to avoid any interreference with normal development. A transgenic line that drives the expression of DsRed under the ubiquitin promoter was generated. Ten sgRNAs that target the 10 unique sequences present in the transgenic lines were injected together with Cas9 protein in one-cell stage zebrafish embryos. Embryos were collected at different time points, and target regions were amplified using primers containing unique molecular identifier (UMI) to add UMI barcodes in the amplicons. (The process of UMI tagging helps in assigning individual sequencing reads back to the cell of origin). Sequencing confirmed the *in vivo* allelic diversity, and the recovered alleles were used to reconstruct the lineage tree. To investigate whether these barcodes can also be recovered in adult animals, several organs (brain, eyes, intestine, gills, heart, and blood) were collected and subjected to DNA sequencing to recover barcode information. It was concluded that most cells in different adult organs were derived from fewer embryonic progenitors; more than 98% of circulating blood in an adult zebrafish contains five common alleles, suggesting a highly clonal origin of the blood system in zebrafish. The GESTALT method was further modified by combining single-cell RNA sequencing to develop scGESTALT [single cell Genome Editing of Synthetic Target Arrays for Lineage Tracing; (Raj et al., 2018)]. The workflow for the cell lineage tracing and scRNA-seq experiment involves the introduction of sgRNAs to target exogenous sequences and the isolation of single cells at appropriate time points. This is followed by mRNA isolation, reverse transcription and cDNA amplification, library preparation, and sequencing both DNA and RNA; this method has been used to identify more than 100 different cell types during brain development. The scGESTALT method also allows barcodes to be recorded at post gastrulation stages by employing temporal control of Cas9 using a heat shock promoter and constitutively expressing sgRNA from the U6 promoter (Raj et al., 2018).

Unlike GESTALT, LINNAEUS, and ScarTrace approaches take advantage of existing transgenic lines carrying multiple integrations of a transgene – green fluorescent protein (GFP) or mCherry. Both LINNEAUS and ScarTrace combine lineage tracing with identification of cell types by single-cell transcriptomics. ScarTrace uses a zebrafish line carrying eight in-tandem copies of a histone–GFP transgene. sgRNA targeting the GFP with Cas9 protein is injected into one-cell stage embryos. Cas9 induces double-stranded breaks that when repaired by non-homologous end joining leave insertions or deletions (scars). During embryonic development, cells accumulate these scars and pass them on to future generations. When the scars are then sequenced, any adult cells containing identical scars must originate from a common progenitor cell. This method also defines cell types based on their transcriptome, thus cataloging both cell type and progenitor for each organ type. ScarTrace revealed that hematopoietic cells in the kidney marrow originated from fewer embryonic progenitors, and multiple progenitors give rise to specific cell types in the brain and eye. It was further revealed that a common progenitor produces both epidermal and mesenchymal cells of the caudal fin. Interestingly, this technique also showed how a progenitor cell commits to produce a left or right eye in zebrafish.

The LINNAEUS approach is similar to ScarTrace, however, LINNAEUS uses a zebrafish line carrying an RFP transgene with 16–32 independent integrations in the genome. The presence of independent integrations in different loci protect the scars from being removed or overwritten by Cas9. sgRNA targeting RFP and Cas9 were together injected into one-cell stage embryos; since RFP-targeting sgRNA generates indels (scars) and leave RFP non-functional, loss of RFP was used as a quality control to monitor the efficiency of editing and scar formation. At later time points, embryos were dissociated into single cells and RFP transcripts were sequenced to quantify the scar formation and the transcriptome from the same cell was sequenced by scRANA-seq. Spanjaard and colleagues applied this approach to identify many different cell types from dissected adult organs including heart, liver, primary pancreatic islets and telencephalon. They found that immune cells from different organs can be grouped together in the lineage tree: analysis of cardiac and pancreatic cell types showed the early separation of myocardial and endocardial lineages.

MEMOIR method uses two different tools: Sequential single molecule Fluorescence *In Situ* Hybridization (smFISH) that reveals which specific genes are active in a particular cell, and CRISPR/Cas9 that generates indels (Frieda et al., 2017). MEMOIR uses bipartite genetic recording elements called barcoded scratchpads. Each scratchpad contains 10 repeat units, and sgRNAs and Cas9 targeting scratchpads induces indels. There is also a barcode adjacent to each scratchpad which can be identified by smFISH and allows for the recording of each lineage. This method has so far only been used *in vitro* to record the cellular history of mouse embryonic stem cells through multiple generations (Frieda et al., 2017).

The methods described above generate complex lineage trees using scarring accumulated over several hours, and each method has a limited number of scars. To overcome this limitation, a self-targeting homing guide RNA (hgRNA) system was developed that can induce scarring over a longer time period and further increase complexity (Perli et al., 2016; Kalhor et al., 2018). Lineage tracing in mammals has been challenging compared to that in zebrafish. Kalhor et al. (2018) used hgRNAs to generate a mouse model for the study of cell lineages during early mouse development; hgRNA containing a targeting sequence with PAM was attached to a scaffold and allowed Cas9 to target the expression cassette. For *in vivo* cell lineage tracing, a transgenic mouse harboring 41 different homing guide RNA expression cassettes was created. This transgenic mouse was bred with a Cas9 expressing mouse strain to induce indels (barcodes). These barcodes can be used to track cells temporally and spatially (Kalhor et al., 2018).

These proof-of-principle studies have developed elegant lineage tracing strategies to establish lineage relationships and understand the fundamental mechanisms of cell differentiation under normal and pathological conditions (e.g., cancer metastasis) in complex model organisms including zebrafish. The development of CRISPR based lineage tracing methods is well-timed and complementary to the efforts toward building the human cell atlas (Regev et al., 2017). These approaches will have significant impact on our understanding of the origin of each cell type and how the adult body is developed from a single cell.

## Conclusion and Future Perspectives

As described above the focus of recent research has been on developing strategies to improve Cas9 function, targeting coverage, and on-target efficiencies by reducing off target editing. CRISPR-based genome editing technologies have revolutionized biological research; CRISPR-related nucleases have been repurposed in many applications, and recent developments in base editing and lineage tracing have further increased their utility in studying development and human diseases. New and inexpensive sequencing technologies are accelerating the discovery of candidate disease genes and pathogenic variants. CRISPR has provided a variety of tools to precisely modify the genome in a targeted manner for a variety of applications including functional gene knockouts, targeted induction or correction of single point mutations, and epigenome editing. Recent work has been focused on refining the specificity and expanding the target coverage of Cas9; directed evolution has led to the discovery of multiple Cas9 variants that will significantly expand the targeting coverage. Furthermore, development of base-editing techniques is an important milestone in the study of pathogenic variants in animal models; they will not only accelerate the functional validation of candidate disease genes in a model organism, but also accelerate the development of therapeutic tools for the treatment of a wide range of human diseases. Development of CRISPR-based lineage tracing methods are revealing information that could have been challenging to uncover using traditional approaches including the discovery of novel cell types and the origin of cells in different organs and tissues in complex model organisms.

## Author Contributions

KL, CP, TR, PV, and GV researched the data. GV wrote the article. All authors read the article and approved it for publication.

## Conflict of Interest Statement

The authors declare that the research was conducted in the absence of any commercial or financial relationships that could be construed as a potential conflict of interest.
